# A multiple species, continent-wide, million-phenotype agronomic plant dataset

**DOI:** 10.1038/s41597-021-00898-8

**Published:** 2021-04-23

**Authors:** Saul Justin Newman, Robert T. Furbank

**Affiliations:** 1 ARC Centre of Excellence for Translational Photosynthesis, Research School of Biology, Australian National University, Canberra, Australia; 2 Biological Data Science Institute, Australian National University, Canberra, Australia

**Keywords:** Plant sciences, Agriculture

## Abstract

A critical shortage of ‘big’ agronomic data is placing an unnecessary constraint on the conduct of public agronomic research, imparting barriers to model development and testing. Here, we address this problem by providing a large non-relational database of agronomic trials, linked to intensive management and observational data, run under a unified experimental framework. The National Variety Trials (NVTs) represent a decade-long experimental trial network, conducted across thousands of Australian field sites using highly standardised randomised controlled designs. The NVTs contain over a million machine-measured phenotypic observations, aggregated from density-controlled populations containing hundreds of millions of plants and thousands of released plant varieties. These data are linked to hundreds of thousands of metadata observations including standardised soil tests, fertiliser and pesticide input data, crop rotation data, prior farm management practices, and in-field sensors. Finally, these data are linked to a suite of ground and remote sensing observations, arranged into interpolated daily- and ten-day aggregated time series, to capture the substantial diversity in vegetation and environmental patterns across the continent-spanning NVT network.

## Abstract (summary)

**Table:** 

Measurement(s)	grain yield trait • protein content • days to flowering trait • Glucosinolate • plant structure development stage
Technology Type(s)	specimen harvest quantity • Mass Spectrometry • agronomic process
Sample Characteristic - Organism	Pisum sativum • Secale cereale x Triticum aestivum • Triticum aestivum • Brassica napus • Avena sativa • Lens culinaris • Cicer arietinum • Lupinus angustifolius • Vicia faba
Sample Characteristic - Environment	cultivated environment
Sample Characteristic - Location	Australia

Machine-accessible metadata file describing the reported data: 10.6084/m9.figshare.13709956

## Background & Summary

Agronomy has an enormous potential to benefit from the diverse and rapidly developing field of statistical methods falling under the ‘machine learning’ (ML) umbrella. Despite calls for greater public data availability^1^, however, the ‘big data’ required to develop and test novel agronomic models and drive a public revolution in the prediction of crop behaviour and management remain largely absent from the public sphere^1^.

Over 500 million smallholder farms, dominantly run by women in the developing world, supply eighty-four percent of the global food supply by value^2^ and contribute the primary source of income for 2.5 billion people^3^. A concerted, open, public effort to observe and predict crop behaviour^4^, using any and all available tools such as machine learning and remote sensing data, can help these people meet the enormous challenges of climate change^5–7^. To achieve such goals, however, requires the provision of open data to the most scientists from the most diverse backgrounds possible.

**Fig. 1:**
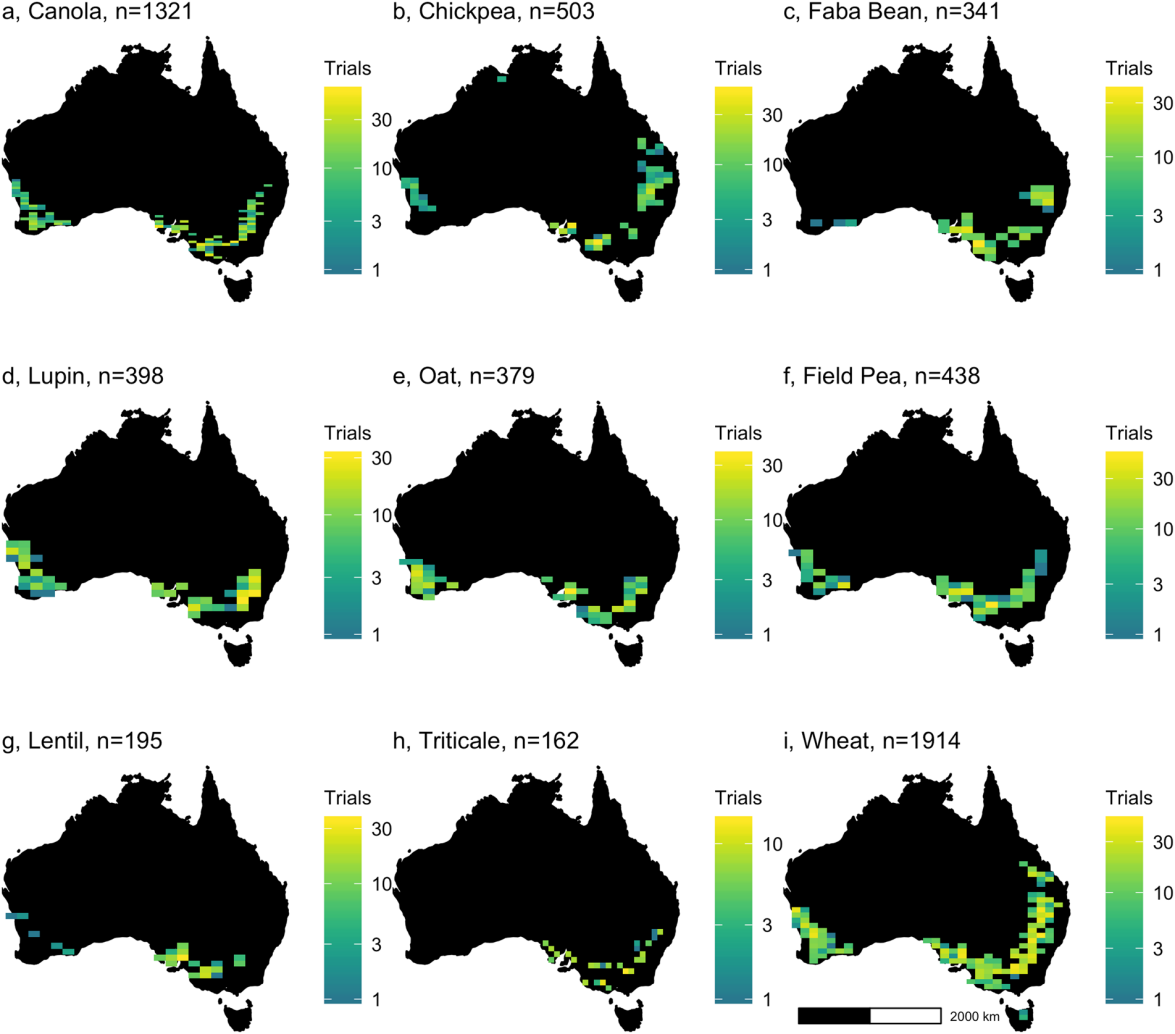
Geographic extent of the National Variety Trials for nine key crops. Density of field trial experiments (**a**–**i**) in the National Variety Trials. Sample size indicates total number of experiments, each containing multiple populations, conducted in each region.

**Fig. 2:**
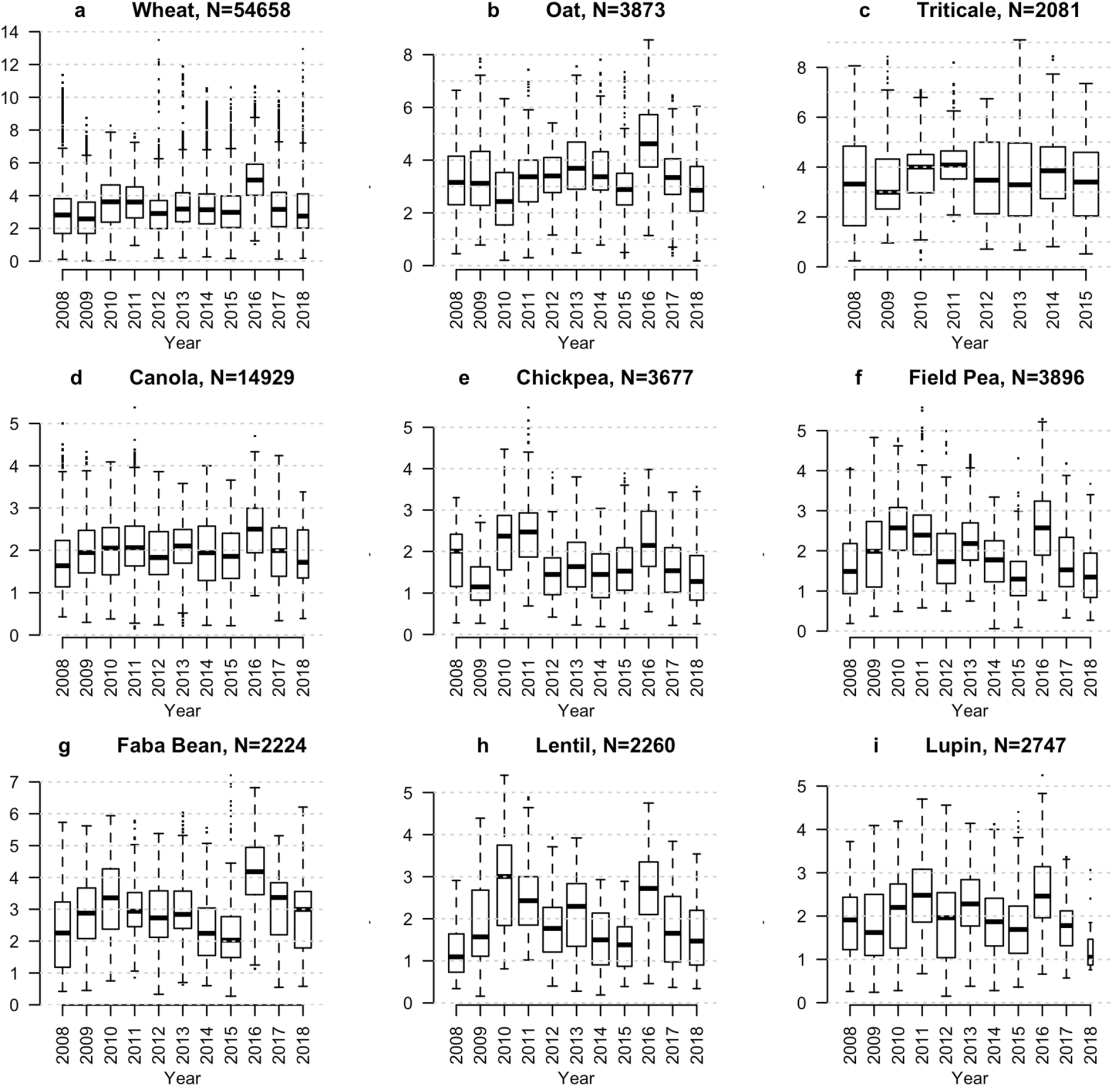
Yield variation in key crop species across the National Variety Trials. Yield in tonnes per hectare across nine key crop species (**a**–**i**) observed in the National Variety Trials. Each point represents a unique variety-site combination, and represents multiple aggregated experimental replicates.

Here, we have assembled integrated continent-wide agronomic datasets (Fig. 1) as a template to help redress this problem. These data contain of over a million phenotypes nested in a quarter of a million crop populations sown across the Australian continent. Key agronomic traits include flowering times, protein content, and over a hundred thousand variety-years of yield data (Fig. 2). By running extensive missing-data imputation, digitisation, and quality controls, and linking these trials to extensive satellite and ground sensing observations, we have formatted these data for the rapid development of machine learning platforms and the testing of agronomic hypotheses on a scale previously inaccessible to other scientists.

To further stimulate the development of agronomic models and the rapid multi-environment testing of fundamental hypotheses these data are used in a related work^8^, in which complex agronomic traits such as yield (R^2^ = 0.8), flowering time (R^2^ = 0.5), grain protein content (R^2^ = 0.5) and Canola Glucosinolate content (R^2^ = 0.6) are predicted using a suite of explainable machine learning models.

These data have clear potential to benefit Australian production through the accurate forecast and understanding of agronomic systems and traits. Optimal variety selection, flowering times, and sowing times are all key drivers of agricultural yield in Australia^9–11^: ML-ready data allows the more accurate generation and retraining of models to predict these factors. In addition, ML models allow the discovery of novel features and patterns predictive of yield and agronomic traits, insights that can be leveraged for Australian grain management and breeding.

However, the greatest value of this analysis is not providing an immediate and definitive answer to agronomic problems, but as an illustration of the benefits of competitive and reproducible ML analyses using open data. These results have been provided in the hope they may be rendered obsolete: by more accurate models, more interpretable algorithms^12,13^, and more imaginative analyses.

We anticipate that provision of the freely accessible ground-truthed data described here will drive innovation in agronomic science, and lead to advances in our understanding of crops through the sphere of public research. While these data will directly benefit the large-scale and industrial farming prevalent in Australia, we hope their provision as a template for success and development may illustrate the enormous potential of greater funding for equivalent, open, unified trial networks across developing nations. Public satellite and weather data remain relatively equitable across the developed and developing world and, for now, leading-edge ML algorithms remain open, accessible, and free. Integrated and expanded ground-truth field trial data, the missing piece required for accurate and global crop models, must be seriously addressed.

Unfortunately, public plant science funding is both meagre^14^ and in rapid decline. Doubling the budget of the International Rice Research Institute^15^, for example, would cost less than three miles of US interstate highway^16^: an absurd funding position, given rice provides a fifth of the global calorie supply^17^. Equivalent shortfalls exist for other crops and trial networks, despite the enormous benefits of plant breeding initiatives^18^. To capture the revolutionary benefits of ML and satellite data for the global poor, these funding positions must improve.

## Methods

These methods are a precursor to the analysis of these data in our related work^8^, with a marginal overlap in the description of these data between works.

### Trial data

Data for 780,569 field trial plots, aggregated by variety into 266,033 unique variety-trial combinations across 8,140 fully randomised controlled yield experiments, were downloaded from the Australian National Variety Trials website across the 2008–2018 field seasons. Any person wishing to use National Variety Trials (NVT) data must request the data directly from GRDC, noting that terms and conditions may apply in respect of usage. These data represent all unique combinations of location, sowing date, and variety of plant population to achieve a non-zero yield or non- ‘failed’ status trials, with trial failure determined by GRDC protocols^19^.

All field trials are run using balanced, replicated, and fully randomised controlled designs. Field trial experiments contain a minimum of two biological replicates of each genotype at each site. In addition, NVT populations are machine-sown to achieve fixed plant densities, for example the 100 to 225 plants per m^2^ achieved in wheat trials^19^. Although seeding densities may be variable across providers, seeding rates are targeted using self-reported viability rates of supplied seed to ensure mature plant densities are constant within trials^19^. The use of machine sowing, phenotyping, and harvesting across trials ensures uniform experimental layouts with consistently low rates of error in layout and phenotypic measurements.

Trial data are published diversely as mixed-format pdf data tables and forms containing metadata, management and phenotypic data, tables of field trial layouts showing the geographic location, relative position, and orientation of varieties within a trial, and “results” data sheets in excel indicating core phenotypes of yield, grain size, and protein content. Examples of each of these formats are available in the file directory provided (file paths /data/trial_results, /data/trial_plans and /data/trial_metadata/originals respectively).

### Phenotypic data

Phenotypic measurements in the NVT are conducted according to fixed standardized protocols across all sites^19^. Core phenotypic measurements are taken using automated and mechanised standard protocols for: yield (N = 104,462 non-zero variety-years; N = 135,582 total variety-years), thousand-grain weight (N = 122,581) and hundred-grain weight (N = 3,719), hectolitre weight (N = 148,768), grain protein fraction (N = 160,020), 2 mm − 2.5 mm sieve fraction (N = 145,401), Glucosinolate content (N = 12,460; Canola only), oil content (N = 14,392), and height (N = 11,105). Another 61 phenotypic measurements were captured in the trial metadata. These included agronomically important phenotypes scored by professional agronomists according to standard protocols, such as: days to 50% flowering (N = 15,248 observations), days to anthesis (N = 1,941), days to heading (N = 7,482), grain brightness (N = 4,017), Zadoks growth scales (N = 7,213), establishment (N = 36,453), early growth rate (N = 26,341), patchiness (N = 3,646), pod shattering (N = 7,502), tillering (N = 6,252), and lodging (N = 15,026) scores.

A total of seventy phenotypic traits were available across the NVTs. Flowering times were reported as a mix of Julian days (from the start of the year) and days after sowing. As quality control, flowering times were corrected relative to the time of sowing and all plots flowering < 30 days after sowing (1983 variety-years; 13% of phenotypes), which were largely flowering time decimal scores (1442 variety-years; 9.5% of phenotypes), were removed. Reported yields included a large proportion of values reported as hyphens, indicating insufficient yields for reporting (23%; N = 31,562). As these were not necessarily zero yields, such yield observations could either be coerced to zero to generate a zero-inflated distribution, or omitted from model training. As entire trials with insufficient yields were already omitted via a similar and unavoidable reporting bias, here the latter approach was used for model training and reporting, and processed data with and without zero-inflated counts are included.

### Environmental data

Data for each NVT location was obtained from the National Aeronautics and Space Administration (NASA) using the online Application for Extracting and Exploring Analysis Ready Samples (AppEEARS), courtesy of the NASA EOSDIS Land Processes Distributed Active Archive Center (LP DAAC), USGS/Earth Resources Observation and Science (EROS) Center^20^ (Sioux Falls, South Dakota, https://lpdaacsvc.cr.usgs.gov/appeears/).

Gridded, processed and orthocorrected data for the MODIS Terra and Aqua satellites and the VIIRS instrument^21,22^ were obtained from both raw spectral bands and diverse processed and composite data products^23–26^. Full lists of these data products are given in the file directory under “DAAC_sample_request.rtf”.

Land surface temperatures of undetermined quality, as flagged by the MODIS post-processing algorithms, were removed^19^. Remote sensing environmental data was converted into daily time series using linear interpolation for continuous data, and last-value-carried-forward interpolation for discrete factorial data^19^. Values falling outside the observed interval, trailing either at the start or end of time series, were imputed at the value of the closest observed datum in the time series. Time series of these variables were constructed relative to sowing dates at each site and time series taken from 90 days before to 275 days after sowing. All land surface temperatures and thermal emissivity observations (bands 31 and 32) not computed due to cloud cover^20^ or other causes such as dead sensor pixels, were removed and backfilled by linear interpolation, with leading and trailing observations respectively filled by last observation carried forward or first observation carried back imputation.

Several additional variables were captured, but not fully featurised. Data on the occurrence of fire events per month were categorised over the previous 5 years prior to sowing using the MODIS data product MCD64A1. In addition, satellite data was used to capture the in-season cumulative total number of days (only) of the following: land surface water (“water_mask” variable; product MOD13Q1_006), frozen surface/ice/snow events (“Snow_ice” variable; product MCD15A3H_006), average or high atmospheric aerosol density events (“Aero_q” variable; product MCD15A3H_006), and days with cirrus cloud cover (“Cirrus” variable; product MCD15A3H_006).

Historical weather data, constituting daily-resolution time series of rainfall (mm), maximum and minimum temperatures (degrees C), and incident solar radiation (MJ/m^2^), were downloaded from the nearest ground station^19^ using the “bomrang” package^27^ in R. Missing data was relatively common during the observation periods, most often caused by the closest weather station missing an instrument, being a weather radar with a station ID but no instrumentation, or by the station being installed or decommissioned during the observation period. Weather stations with more than 25% of data missing were excluded, and observations from the next-closest available station used^19^.

Both the weather station data and remote sensing data time series were featurised by aggregating all variables over 10-day blocks from 90 days before to 270 days after sowing. The geometric mean, variance, maximum, minimum, and cumulative sum (for non-negative variables), were calculated within each consecutive 10-day block for all variables for use as features. For net photosynthesis (MODIS product MOD17A2H_006), a constant of 0.2 was added to all values to prevent addition of negative values in cumulative doses of photosynthesis. In addition, to account for any effect of sowing date in the Australian wheat environment^10^, the number of days to sowing from the start of the year (‘Julian days to sowing’) was included as an additional variable.

### Management and metadata

Metadata were scraped from field trial reports in 6,547 of the non-failed field trial locations. These extensive metadata include on-site observation of soil test data, pathogen severity, drought severity, daily frost incidence, rainfall, temperatures, diverse phenotype scores, flowering dates, animal damage^19^. These in-field metadata data also include in-field management histories spanning 5–6 years prior to sowing. These management histories include data on the brand, timing, active ingredient, dosage and per-hectare dosage of both fertilisers and pesticide applications, and the species sown in previous crop rotations.

Given the mixed reporting of doses all pesticide doses applied within the trial year were recalculated to g/Ha of active ingredient, with the exception of a limited number of applications of the nematicide Iprodione which were converted to equivalent “seed” doses. Due to infrequent reporting of doses and a high frequency of outlier (order of magnitude error) doses, pesticide application histories over previous years were recoded as to indicate the presence or absence of application only.

Both previous and in-season fertiliser doses were recalculated into kg/Ha of product applied. In addition, total Nitrogen, Sulphur, and Phosphate in kg/Ha was calculated for each application.

### Constructed variables

We calculated a limited number of variables using targeted domain knowledge. Given the potentially dominant importance of sowing timing for crop yield in Australian contexts^10^, an integer ‘Julian days to sowing’ variable was created to capture the number of days from January 1 of each year to the sowing date. Two binary-coded variables were constructed, a variable to discriminate between dicots and monocots, and a variable indicating membership in the Fabaceae to capture the functional role of Nitrogen fixation in crop yield. A logical variable was generated to indicating if the prior crop rotation was the same or a different species, to account for potential undetected alleleopathic or accumulative yield effects.

### Quality control, recoding, and imputation

Metadata variables were most likely to have high (over 10%) rates of missing data. Frequently these data constituted observational scores, such as specific animal damage or grazing scores, that were infrequently measured in a trial context.

Extensive error correction was applied to handle the collective data entry errors of field annotations from agronomists such as the eight common spelling variants on “Trifluralin”, the six common spelling variants of “Granulock 12Z”, or the frequent insertion of asterisks, escape characters, and extensive field notes into single spreadsheet cells. These extensive and mixed format and spelling errors resulted in coding numerous low-frequency variety names as “unspecified”.

Dummy-coding was performed for factors with a high number of levels. Varieties observed in over 1,000 experiments, and free-form metadata comments applicable to over 1,000 variety-experiment combinations (*e.g*. “replicates 2–3 unsprayed by fungicide”), were dummy-coded for presence or absence. Pesticide and fertiliser applications with known application to a field, but unknown or incorrectly recorded doses, were encoded as “NA” (not applicable) values to discriminate from known zero doses. Applications of multiple fertilisers or pesticides with identical dates were aggregated by active ingredients. Pesticides applied to less than 10 experiments were removed, as they accounted for less than twenty percent of all observations but over ninety percent of unique pesticide names. Likewise, fertiliser names observed in less than ten experiments were removed.

**Fig. 3:**
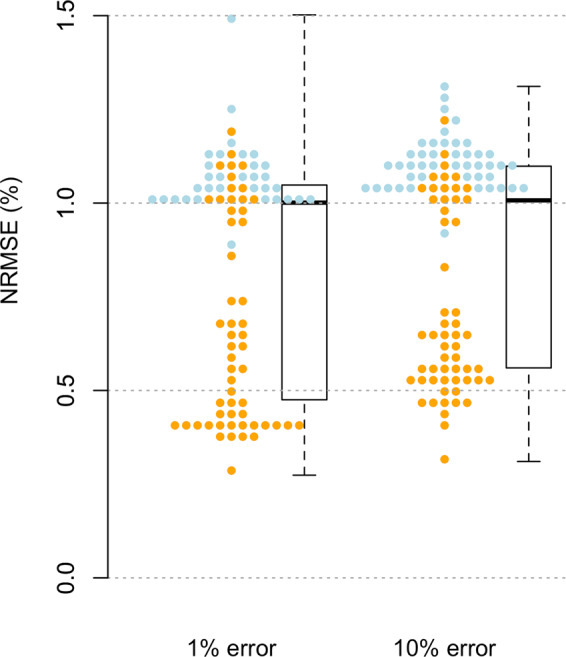
Imputation accuracy for predicting simulated random errors. Random errors at one and ten percent frequency, introduced into a random-sample imputation, reveal relatively low normalised root squared mean error (NRMSE) rates of random forest imputation when applied to data that are missing completely at random. Points indicate average NRMSE into column-wise chunks^19^ with a bimodal distribution of error rates resulting from imputed weather station data (blue).

Despite a low rate of missing environmental data after quality controls (0.7% missing overall; 0.05% missing satellite data), list-wise deletion could not be achieved without near-complete loss of observations given the substantial size of the dataset. Instead, missing data were imputed using two approaches. Non-phenotypic missing data were imputed for the entire dataset across 25 independent random-sample imputations, in which randomly selected data are used to in-fill missing values, as an unbiased but lowest-accuracy imputation method. Second, missing data were imputed another 25 times using random forest equations, trained to in-fill missing variables, using the ‘missForest’ package^28^ in R. Random forests under the missForest algorithm were run with five maximum iterations, 25 random-sampled variables per tree, and ten trees, run across ten nodes with parallelized variables. As data were sorted by domain and time series across columns, imputations were chunked into 300-column groups of variables to increase computation rates. Associated imputation error rates are presented in Fig. 3.

## Data Records

The data have been provided within a single compressed directory structure (available from figshare^19^) containing the source NVT data from which the complete datasets are assembled (“/data/”), satellite data in the “MODIS_data” directory (or is freely available from the National Aeronautics and Space Administration through the LP DAAC^20^), and both a heavily annotated R script and an R workspace used to compile the complete dataset (the.rdata and.r files). Linearly interpolated daily time series data is provided for all satellite sensor products, at all sites during a given year, in flat.csv text files under the “data/constructed_csv_files/Satellite_ts_YYYY.csv” file paths^19^. In addition, the completed dataset itself is provided, both as multiple imputations of the whole-experiment data (the “~/site_imputations/filename.rds file paths), and as flat text files for all sites and for all unique variety-site combinations (the “/model_frames_and_imputations/variety”.rds and.csv files) for the first random forest based imputation only^19^.

The data follow a variable naming convention (Supplementary Tables 1–2) that contains a data domain, such as the prefix “SAT” for all satellite-derived variables (Supplementary Table 1). Variables within a time series or with a time component then contain either a suffix indicating the timestamp of the data, relative to the day of sowing at time 0, or a categorical flag such as “date” for exact dates or “post_sowing” for data derived at an unknown date after sowing (*e.g*. harvest index measures). Unless explicitly given as calendar or R base format numeric dates^19^, all times are given relative to the time of sowing at *t = *0.

## Technical Validation

To test the accuracy of field site GPS data, all of the 2018 field site GPS locations were manually checked for accuracy, using publicly available remote sensing data embedded in google maps. No errors resulted, however this approach could not be extended to previous years given the sampling and cost limitations of high-resolution satellite data.

Several of the phenotypes consist of scored variables, such as disease severity scores, or occasionally a mix of ordered factor scores and numeric observations. For example, flowering time data included a small fraction data scored 1–10, while the remainder consisted of either a date or the number of days to flowering. While a quality-controlled version of some of these variables has been included, such as the removal of 1–10 flowering time scores to create the “PHENDom_flowering_QC” variable, some issues may remain with lower-frequency phenotypes.

A considerable effort has been expended in correcting spelling mistakes, carriage returns, field entry errors, unexpected escape characters, formatting errors, mislabelling, and the incorrect insertion of extensive commentary into data fields. Extended detail, running to hundreds of lines of code, is available in the annotated R code. However, errors will undoubtedly remain. Particular care should be taken with the mix of typographic errors, mixed brand names and types, re-branding of identical products, partial re-formulation of products with identical names, and other assorted problems apparent in the roughly 350,000 pesticide and fertiliser applications. In addition, the naming of less common or unreleased plant varieties often follows coding conventions that employ alphanumeric codes (*e.g*. wheat varieties such as “ADV11.9419” and “LPB14-0392”) that do not allow screening of typographic errors. Finally, the assumed homogeneity of plant varieties is often not borne out by genomic or phenotypic indicators. Even nominally selfing species such as wheat can out-cross under stress, retain heterozygosity across many generations, or become accidentally mixed with other genotypes. For example, Wyalkatchem and Yitpi wheat varieties were respectively flagged or removed from all 2011 NVTs in Western Australia, as they contained obvious heterogeneous mixes of unknown origin.

A substantial fraction of the satellite data used here are from the paired MODIS Terra and Aqua satellites, which ensure sensor and measurement redundancy, subjected to intensive and standardised quality controls described in detail in the NASA LP DAAC documentation^20^. The Australian Bureau of Meteorology data are implemented as-is, despite for example misspecification of some weather station locations (*e.g*. the Wyening station).

## Usage Notes

These data represent an aggregated non-relational database compiled by diverse agronomists working as government employees or subcontractors, across an entire decade, and as such due caution is required for the interpretation and understanding these data. In addition, all phenotypic data, metadata, management data, and some environmental data were extracted from excel files with mixed escape characters and errors, flat pdf files, and cached internet files. While a considerable effort has been made to correct errors and develop a consistent data format, as in all datasets caution should be exercised to screen outliers and errors.

The potential role of missing-not-at-random data can be addressed in factorial data by treating null, missing, or “NA” data as a unique factor, as we have done. However, numeric data is more complex. Due to the large scale of data here, missing data should be subjected to extensive nonparametric testing^29,30^ to assess whether it satisfies criteria for reliable imputation. Here, we have estimated noise from imputation errors by assuming errors are distributed completely at random^31,32^, with the corollary assumption that data missing from the GRDC data are likewise missing at random. While a common and often necessary assumption, this is not a safe assumption^31^. As discussed elsewhere^8^ there are phenotypes in this data that are not missing at random, such as zero-yielding trials, and the potential role of non-randomly distributed missing data in model development requires careful assessment on a case-by-case basis.

Immediate use cases for these data include the development of ML models to predict variation in key agronomic traits such as yield and protein quality, the prediction of developmental milestones such as flowering in response to environmental shifts, and the differing behaviour of genotypes and species across environments. For example, the better pre-sowing prediction of yield allows for more varietal choices at the time of sowing. Dynamic predictions updated using data feeds from remote sensing data represent another potential target. For example, to prevent substantial losses at anthesis to either frost damage or heat-induced sterility, sowing times and phenology have to be optimally targeted within a ‘goldilocks’ seasonal temperature range^33,34^. Better prediction of varietal flowering times through ML has the potential to dramatically reduce such temperature-induced losses, without requiring knowledge of model mechanics: all that is required is the empirical prediction of optimal sowing times for a given variety or species.

Such models are open to dramatic improvement through reproducible and open competition. Indeed, our analyses of these data^8^ have been conducted in the hope that our models will be rapidly superseded and expanded. The potential for ML models to generate explainable models^12,13^ or integrate variety-specific genomic and epigenomic data are particularly exciting areas for agronomy. However, open data is not limited to known challenges. The potential uses of this trial data may be best viewed as a set of ‘unknown unknowns’ previously stifled, not by a lack of imagination, but by a lack of access.

## Supplementary information

Supplementary Tables

## Data Availability

All data and code is available without restrictions from figshare^19^ and from the corresponding author on request.
