# Author Correction: A multiple species, continent-wide, million-phenotype agronomic plant dataset

**DOI:** 10.1038/s41597-022-01536-7

**Published:** 2022-07-22

**Authors:** Saul Justin Newman, Robert T. Furbank

**Affiliations:** 1 ARC Centre of Excellence for Translational Photosynthesis, Research School of Biology, Australian National University, Canberra, Australia; 2 Biological Data Science Institute, Australian National University, Canberra, Australia

**Keywords:** Plant sciences, Agriculture, Plant sciences, Agriculture

Correction to: *Scientific Data* 10.1038/s41597-021-00898-8, published online 23 April 2021

The authors wish to publish the following disclaimer relating to the use of data from the Grains Research and Development Corporation (GRDC) in the dataset associated with this article, following concerns raised by GRDC:

*This dataset is based predominantly on data sourced from Grains Research & Development Corporation (GRDC) and GRDC’s extensive investment in the collection, development and presentation of this dataset is acknowledged*.

*GRDC did not authorise the reproduction, publication or communication of this dataset and this dataset has not been subject to GRDC’s quality control processes, does not include updates and corrections that have been made to this dataset and as such may be unreliable, and results of research based on this dataset should not be relied on for any purpose*.

*Any person wishing to conduct research using National Variety Trials (NVT) data must approach GRDC directly with a research proposal, noting that terms and conditions may apply*.

*Scientific Data* wishes to alert readers that the reference to the access terms for the underlying GRDC data used to create this dataset was not accurate in the original publication. This information has been updated in the “Trial Data” section of the manuscript. The figshare dataset associated with this Data Descriptor remains available under a CC-BY licence, hence this correction does not restrict readers’ use of the figshare data beyond the original terms.

