# Supplementary material for: A multiple species, continent-wide, million-phenotype agronomic plant dataset
Source: Sci Data. 2021 Apr 23;8:116. doi: 10.1038/s41597-021-00898-8 (PMC8065040; doi:10.1038/s41597-021-00898-8)
Supplement: Supplementary file 1 — Supplementary Tables [file 41597_2021_898_MOESM1_ESM.docx]

**Table of Contents**

**Page 2 – Supplementary Table 1. Variable naming guide: data domains**

**Page 3 – Supplementary** **Table 2. Variable naming guide: time series labels**

**Supplementary** **Table 1. Variable naming guide: data domains**

| Variable name component | Description | Example | Interpretation |
| --- | --- | --- | --- |
| **Domain Prefix** | |  |  |
| PHENDom_ | Phenotypic data | PHENDom_Flowering_50pct | Days after sowing until 50% of plot flowering |
| METADom_ | Trial metadata derived from PDFs | METADom_AMMONIUM_  SULPHATE | Dose of Ammonium Sulphate per hectare in g/Ha |
| MANDom_ | Management data | MANDom_Rows_per_plot_6 | Trial was sown with six rows per plot |
| BOMDom_ | Bureau of Meteorology data | BOMDom_solar_exposure | Daily solar irradiance at nearest weather station |
| SatDom_ | Satellite-derived data | SatDom_LAI | MODIS-estimated leaf area index |
| ENVDom_ | In-field environment data | ENVDom_Frost_Damage_score | Agronomic frost damage score out of 10 |
| **Subdomain** | |  |  |
| _Fert_rotation_ | Fertiliser rotation metadata | METADom_Fert_rotation_minus_1_N | kg/Ha of applied Nitrogen -1 year ago |
| _Chem_rotation | Pesticide rotation metadata | METADom_Chem_rotation_minus_2_ATRAZINE | Atrazine was applied -2 years ago |
| _Crop_rotation | Crop rotation metadata | METADom_Crop_rotation_minus_4_sub_cropCanola | In-field crop -4 year ago was canola |
| _10cm.test_ | Soil testing horizon (10cm or 60cm) | METADom_Organic_C_10cm.test_10cm | Total Organic Carbon 10cm deep |
| _Variety_ | Variety name (common varieties only) | METADom_Variety_CALINGIRI | Variety planted was *Calingiri* |
| _Breeder | Varietal breeder | MANDom_BreederNational.Lupin.Initiative | Variety was bred by National Lupin Initiative |
| _Series_name | Trial series name | MANDom_Series_nameAdvMainAgzone6 | Advanced Main Season Trials in Agzone6 |
| _Trial_operators | Company subcontracted to run trial | MANDom_Trial_operatorsSARDI | SARDI-run trial |

**Supplementary** **Table 2. Variable naming guide: time series labels**

| Variable name component | | Description | Example | Interpretation |
| --- | --- | --- | --- | --- |
| **Timing Suffix** | | |  |  |
| _.30 | -30 days before sowing (minus is indicated by period) | | BOMDom_max_temperature_mean_.30 | Mean of maximum temperatures 30-21 days before sowing |
| _100 | +100 days after sowing | | BOMDom_max_temperature_mean_100 | Mean of maximum temperatures 100-109 days after sowing |
| **Timing factor** | | |  |  |
| _time_ | timing of application (relative to sowing date) | | METADom_Fertiliser_time_MAP | Time in days relative to sowing for application of Monoammonium Phosphate |
| _repeat_ | indicates repeat measures/applications | | METADom_DIQUAT_repeat_1 | Dose for second in-season application of Diquat |
| **Aggregated feature** | | |  |  |
| _mean_ | mean over 10 day period | | SatDom_EVI_mean_30 | Enhanced Vegetation Index 30-39 days after sowing |
| _max_ | maximum over 10 day period | | SatDom_EvapoTrans_max_10 | Maximum evapotranspiration 10-19 days after sowing |
| _min_ | minimum over 10 day period | | SatDom_GPP_min_150 | Minimum Gross Primary Productivity 150-159 days after sowing |
| _var_ | variance over 10 day period | | BOMDom_min_temperature_var_90 | Variance in minimum temperatures 90-99 days after sowing |
| _csum_ | cumulative sum | | BOMDom_rainfall_csum_110 | Cumulative rainfall to 110 days after sowing (from 80 days before sowing) |
